# A Randomized, Prospective Study of the Treatment of Superficial Partial-Thickness Burns: AWBAT-S Versus Biobrane

**Published:** 2011-02-24

**Authors:** John E Greenwood

**Affiliations:** Adult Burn Service, Royal Adelaide Hospital, North Terrace, Adelaide, South Australia, Australia

## Abstract

The aim of this study was to prospectively evaluate AWBAT-S in comparison with Biobrane for the treatment of superficial partial-thickness burns using the patient as his or her own control. Primary outcome measures included length of hospital inpatient stay and patient-reported perception of pain. Secondary outcome measures included time to healing, clinical outcome of burn sites (scarring) and a comparison of cost of care for patients treated with AWBAT-S versus Biobrane.

Superficial partial-thickness burns involve damage to the epidermis and superficial dermis.^1^ The destroyed tissue typically blisters and sloughs off leaving an open and exuding dermis with nerve endings exposed. They thus represent one of the most painful of the several categories of thermal injuries. Historically, conservative treatment consisted of removing nonviable tissue on the ward (the aggressiveness dictated by pain), daily bathing or showering with friction washing of burn wounds and applying new dressings with topical medications 1 to 2 times a day.^2^,^3^ These procedures cause severe pain and anxiety in patients, even with the use of opiate analgesics. The management of these injuries at the Royal Adelaide Hospital (RAH) involves aggressive cleaning under general anaesthetic immediately, or within 24 hours, after the burn injury and arrival at hospital, followed by the application of a biosynthetic epithelial replacement (Biobrane, Dow Hickam/Bertek Pharmaceuticals Inc, Sugarland, Texas; distributed by Smith & Nephew Medical Ltd, Hull, UK).^4^ This has been performed with great success in more than 1000 cases over 9 years.^5^

The application and subsequent firm adherence of Biobrane, a partly occlusive dressing, allows reepithelialization to occur underneath and eliminates the need for daily bathing and frequent dressing changes. Although several skin substitutes are available commercially, Biobrane (a biosynthetic wound dressing constructed of a silicone film with a nylon fabric partially embedded into the film) presents to the wound bed a complex 3-dimensional structure of trifilament thread to which collagen has been chemically bound. Serum exudate clots in the nylon matrix (most likely because of conversion of exudate fibrinogen to fibrin after exposure to the porcine type 1 collagen peptides), thereby firmly adhering the dressing to the wound until epithelialization occurs. It has been effective in the treatment of partial-thickness burns since 1982.^6^^-^18

A more recent product, AWBAT-S (Advanced Wound Bioengineered Alternative Tissue – Superficial, Aubrey Inc Carlsbad, California), which is comparable in cost to Biobrane, has been cleared by the US Food and Drug Administration and is commercially available. Although similar in many ways to Biobrane, there are some dissimilarities. Both materials have a thin medical-grade silicone membrane (0.001-in thick), which controls water vapor transfer and maintains a moist healing environment. Both have a fine woven nylon fabric (15/3 denier—Biobrane and 15/2 denier—AWBAT), which gives the skin substitute its strength, elasticity and ability to be surgically secured. Both have pores in the silicone membrane to enable excess fluid/exudate to escape the wound surface through the skin substitute into a sterile outer dressing. This minimization of fluid accumulation adjacent to the wound surface reduces proliferation of endogenous wound bacteria. Fluid accumulation (seroma) also compromises adherence of the skin substitute to the wound surface, which is the most important property of an effective skin substitute. Biobrane has pores in the silicone membrane at ½-in centers. AWBAT-S has pores in the silicone membrane at ¼-in centers. The area of the AWBAT-S pores (8.8 mm^2^) is larger than the pores in Biobrane (6.2 mm^2^) making AWBAT-S approximately 500% more porous than Biobrane. The greater porosity of AWBAT-S is expected to result in improved transfer of fluid/exudate from wound surface to outer dressings, which may result in lower rates of infection, better acute adherence and shorter healing time. Both materials contain collagen peptides for the purpose of reacting with the fibrin in the wound to achieve good acute adherence. In Biobrane, cyanuric chloride (a carcinogen) and dodecylamine (an allergen) covalently bond the collagen peptide to the silicone-nylon composite. AWBAT-S uses no toxic or allergic cross-linking agents in its collagen binding. As there is residual dodecylamine in Biobrane, an allergic reaction mandates removal and prohibits further application. Allergic reactions are rare with AWBAT-S and allergens have not been demonstrated. Biobrane has low levels of immobile collagen peptide (porcine type I at approximately 2.5 µg/cm^2^). AWBAT-S has more collagen peptides of the same type (at approximately 10 µg/cm^2^) and highly mobile, which enables the peptides to quickly react with fibrinogen in the wound exudate and achieve the acute adherence desired. Both materials are provided sterile in a sealed package; Biobrane is sterilized with an autoclave (live steam) and AWBAT-S by electron beam.

## METHODS AND MATERIALS

The study was registered with the Australian New Zealand Clinical Trials Registry and allocated the registration number ACTRON 12609000765224.

### Study population

We intended to enroll all patients admitted with burns expected to heal spontaneously (who would usually undergo aggressive burn debridement under general anaesthesia and Biobrane application). Inpatient treatment over the next 12 months was used for clinical follow-up visits, scar assessment, clinical outcome assessment, and data analysis.

### Study duration

It was anticipated that all subjects would be enrolled and complete their follow-up visits at 12 months post application.

### Inclusion/exclusion criteria

For inclusion, patients had to have superficial partial thickness to mid dermal burns with 2 noncontiguous burn sites of the same approximate size/depth for comparison or 1 burn site large enough to accommodate both a 6-in AWBAT dressing and a 6-in Biobrane dressing. To enable this, the burn wounds had to range between 2% to 40% total body surface area (TBSA). The research ethics committee insisted that the patient age ranged from 18 to 70 years. Exclusion criteria included delayed presentation (>48 hours, Biobrane is never applied after this time in my practice because the burn has “dried out” and cannot be made to appropriately exude after this time), ventilator dependence, non-English speakers (consent impossible), signs of burn wound infection (another indication for nontreatment with Biobrane), pregnancy/lactation, burns of unpredictable early depth and course (electrical, chemical or frostbite injury) and comorbidity which may compromise healing or any known allergy to porcine products.

### Randomization and group allocation

After admission to the Burn Centre, a member of the research team contacted the patient to invite them to participate in the study. Signed consent (both research and standard RAH surgical) was obtained following full explanation of the study. The patient was screened for inclusion/exclusion criteria. The random allocation of dressing locations was by randomization table and sealed envelopes, which were opened in the operating room and the wounds dressed in accordance with the instructions provided.

### Procedures and Assessments

Once the patient was enrolled and consent obtained, a visual assessment and measurement of the burn wound(s) was made by the surgeon and recorded. The surgeon matched sites by approximate size and depth and identified them anatomically (noncontiguous, right or left; contiguous, superior or inferior, medial or lateral). Under general anaesthesia, the burn wounds were meticulously cleaned according to Burn Centre Surgical Protocol which included shaving any hair from the surface of the burn and for 10 cm around the burn to aid Hypafix (BSN Medical GmBH, Hamburg, Germany) adhesion and minimize discomfort.

Using the patient-specific randomization envelope, one burn wound anatomical site was dressed with AWBAT-S and the other anatomical site was dressed with Biobrane. Alternatively, both materials were used adjacently on large burns and their respective positions were randomized. Both AWBAT-S and Biobrane were applied according to the manufacturer's instructions (AWBAT-S could only be applied under moderate stretch to prevent destruction of the 3-dimensional matrix of the skin substitute and compromising both acute and secondary adherence). The dressings were secured with sterile Hypafix tape and covered on the limbs with burns gauze soaked in weak (1 in 10) povidone iodine solution followed by a crepe bandage (limbs). On the trunk, dressings consisted of Acticoat (Smith & Nephew Ltd, Hull, UK) and Exudry (Smith & Nephew Ltd, Hull, UK) (posterior) or Acticoat and Cutilin (DeFries Industries, Melbourne, Australia) (anterior).

The wounds were inspected on day 1 by removing the outer dressings down to the Biobrane/AWBAT-S layer and assessed for their intactness and complete wound coverage, seroma or hematoma formation, signs of infection, pain experienced, exudation, quality of dressing including conformability, pliability, elasticity and any adverse event. Redressing was performed according to Burns Centre dressing protocols and the dressings used were recorded on the case report form.

Adult patients were instructed to assess and report pain as 0 to 1, no pain; 2 to 3, mild pain; 4 to 5, uncomfortable to moderate pain; 6 to 7, distressing to severe pain; 8 to 9, intense to very severe pain; or 10, unbearable pain. The time of administration, dose, and route of any analgesia administered within 4 hours of examination was noted on the case report form.

This process was repeated on day 2 and subsequently as required until healing. After healing and Biobrane/AWBAT-S separation, a scarring assessment was also made at each time point using the Vancouver Scale. Digital photographs were taken at every visit after photographic consent was obtained.

### Data analysis

Statistical significance was determined by 2-tailed student *t*-test for parametric data (time to healing). A 2-tailed *t*-test was also used to analyze the difference (matched pairs) for each day between medians for pain scores. A probability level of 0.05 was used as the criterion for significance.

## RESULTS

The anticipated recruitment for this study was based on the large number of Biobrane applications for superficial partial thickness burns over the previous 7 years. However, disappointingly, a very large number of these injuries presenting within 13 months of the study commencement met exclusion criteria (specifically the inability to understand the study and to provide “informed” consent). After commencement of the study, Aubrey Inc released what they claim to be a more effective epidermal skin substitute (AWBAT-plus). These 2 factors together prompted discontinuation of the study after 14 months. The data produced, however, is important in evaluating the AWBAT platform. The results have been summarized in Tables 1 and 2.

### Length of stay

**Figure 1:**
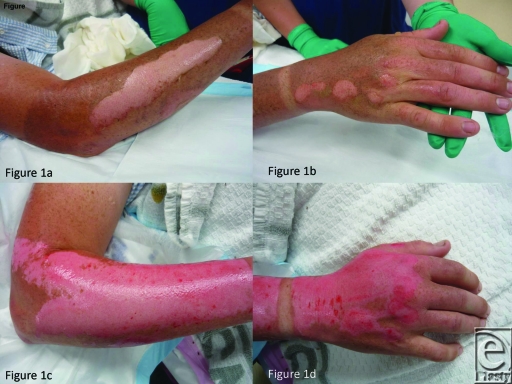

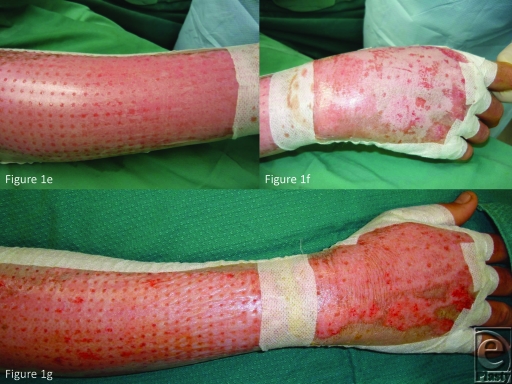

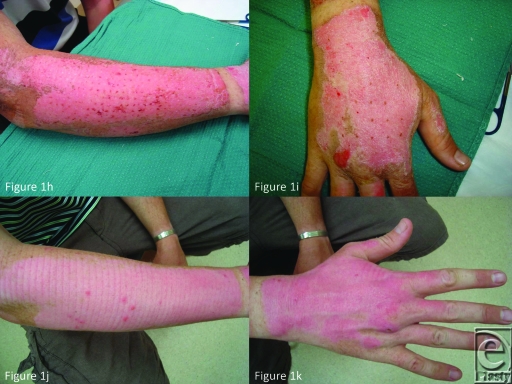

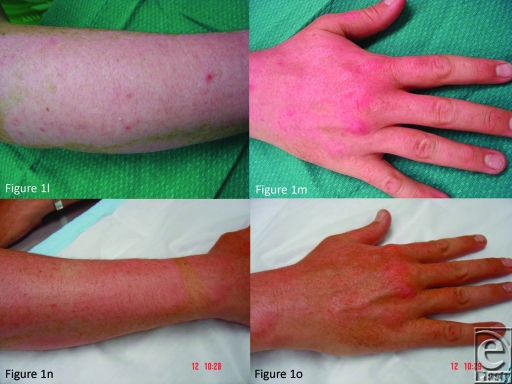
(*a* and *b*) Appearance on presentation. (*c* and *d*) Meticulous cleaning and debridement. (e and f) Materials applied (AWBAT-S to forearm, Biobrane to hand). (*g*) Appearance of both sites at day 3. (*h* and *i*) Both sites healed by day 7. (*j* and *k*) Appearance at day 15. (*l* and *m*) Appearance at day 21. (*n* and *o*) Appearance at day 28.

Generally, the usual length of stay in the Burns Centre at the Royal Adelaide Hospital is 1 day per percent TBSA. This was confirmed during this study (0.88 days per percent TBSA). This is despite the fact that the specialized nature of Biobrane means that some patients (rural dwellers—several hours to several days from Adelaide, those whose compliance cannot be relied upon for any reason) are encouraged to stay longer. In this study, only in 2 patients (with the largest TBSA burns) did the length of stay in hospital exceed the time to healing. Both of these patients were referred from areas 4 to 5 hours drive from Adelaide (Table 1). In one patient, although the burn was smaller (6% TBSA), its site (which included the groin) caused too much discomfort to allow earlier discharge. In all other cases, the presence of Biobrane or AWBAT-S did not prove a barrier to discharge. Figure 1 (*a*-*o*) demonstrates a typical progression from admission to healing in AWBAT-S compared to Biobrane.

### Time to healing

**Figure 2:**
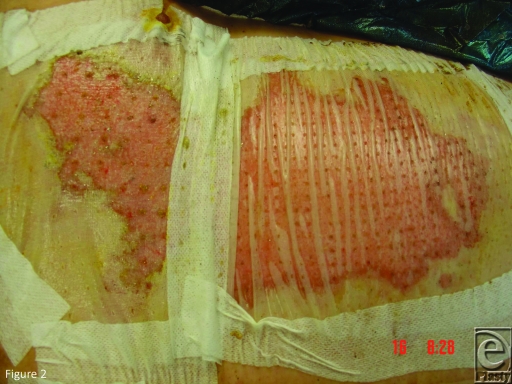
The wrinkling appearance that affected some AWBAT-S in the early stages belies complete adherence.

With small study numbers (n = 12), the mean time to healing between the groups was not significantly different (Biobrane, 10.17 ± 2.33 days compared with AWBAT-S, 9.58 ± 2.39 days, *P* = .09—Table 2). This might achieve significance with larger study groups. There were some features of healing under AWBAT-S, which made the material different to Biobrane, such as wrinkling, which gave the appearance of linear nonadherence and caused concern in a few early patients (Fig 2). This occurred despite the AWBAT-S being applied flat and under tension. It usually settled by day 5. Once these differences were appreciated, AWBAT-S was as easy to manage as Biobrane.

### Pain experienced

Three patients had pain scores that were extraordinarily high in our experience for Biobrane (No. 1, 2 and 12—Table 1). The individual perception of pain experienced by each patient can skew data and this study method of using both materials on each patient (both treatment and control arms being subject to individual pain perception) help to negate such outlying effects. Table 1 demonstrates that the pain scores for both materials in all patients were very similar throughout the study. Pain scores up to day 3 have been included, after this point the pain scores dropped to zero in most patients. There were no significant differences between median pain scores with Biobrane or AWBAT-S at any time point (day 1, *P* = .49, day 2, *P* = .34, day 3, *P* = .38—Table 2).

### Cost of treatment

Subtracting the cost of the material itself (the Australian market cost of AWBAT-S is not yet known), both materials were identical in terms of outer dressing cost, nursing attention, and analgesia. Over the 12 patients, 7 days less treatment was required for wounds dressed with AWBAT-S (although this did not equate to a significant difference).

## DISCUSSION

I personally believe that the development of Biobrane, with its subsequent widespread availability, has been an important step in reducing pain and facilitating therapy in burn injuries expected to heal spontaneously. Those clinicians managing burns who do not use this material in this indication, favoring more traditional conservative approaches, have failed to grasp the magnitude of these patient outcomes (in particular pain) and nursing resource issues (such as a reduction in dressing time). The material is not, however, without its downsides and the meticulousness with which the wound bed must be prepared no doubt disinclines some surgeons to use it. No less meticulous preparation is needed before AWBAT-S application. I would suggest, however, that such wound preparation allows an unparalleled opportunity to decontaminate the wound and fully assess burn depth, degree of exudation and in some cases (where epidermis is critically injured but not detached) even burn size. I have also experienced personally the deleterious effect of “delaying intervention to allow the burn to declare.” In refusing to acknowledge the validity of this claim, and interfering early in the evolving pathophysiological process, I have markedly reduced my grafting rate with concomitant shortening of length of hospital stay and time to return to work, reduction in time to full function and improvements in scarring, and patient satisfaction with cosmetic and functional outcome. My experiences with Biobrane have been published demonstrating my reliance on it in a range of burn situations.^5^ When Aubrey Woodroof revealed his development of a new, biosynthetic epidermal skin substitute, AWBAT-S, claiming that he had ironed out some of the problems associated with Biobrane, I was skeptical and excited at the same time. He claimed that the increased porosity would decrease the incidence of seroma (which I have never experienced with Biobrane but which clearly perplexes others) and that the extension of the matrix across the pores (so that only the silicone layer was deficient at these areas) would reduce the “pore marks” reported by some authors with Biobrane use. He claimed less “allergic reaction” (which again I have not experienced with Biobrane). In actual fact, when one considers my avid loyalty to Biobrane, he was extremely brave to allow me to compare AWBAT-S against it.

Obviously, the outcome of this study in simple terms could only be one of 2; AWBAT-S performs better than Biobrane or AWBAT-S does not perform better than Biobrane. In the case of the former, whether a surgeon changes to a newer material depends also on reliable availability, ease of access and supply (which can depend heavily on the national local regulatory body) and cost.

Biobrane's main advantage over other conservative treatments is its adherence and elasticity (which prevents shear against the wound bed during dressing changes even with joint movement, such as metacarpophalangeal joints, proximal interphalangeal joints and distal interphalangeal joints flexion in making a fist)—these are the properties, which reduce pain and speed return to function. There are a number of “surgical” features where, personally, AWBAT-S is not as good as Biobrane. One such is in its ease of fixation with fixative tapes. The smoothness of the silicone layer lacks the “texture” of Biobrane and Hypafix simply does not stick so well to it. Since my application technique for Biobrane relies heavily on me being able to use tape to stretch and hold the material, this is a major problem for AWBAT-S.^19^ In addition, AWBAT-S is nowhere near as elastic as Biobrane and thus, each piece covers less wound area (which would make each treatment more costly, even if both materials cost the same). The new material does not adhere like Biobrane, remaining “wrinkled” clinically, which can give the impression that it has not stuck. However, this is a false impression, which is dispelled during the first use.

In terms of nursing staff preferences, AWBAT-S is considerably easier and less uncomfortable to remove than Biobrane. Biobrane has 2 problems in this regard—the first is that the material frequent sticks at the pores due to coagulated exudate, the second is that Biobrane usually has a “Velcro-like” attachment to the healed burn away from the pores. The continuation of the nylon matrix across the pores in AWBAT-S removes the first problem. The looser binding of the protein to the matrix in AWBAT-S may be responsible for the wrinkling appearance and seems to make removal far easier.

In terms of patients, all preferred AWBAT-S during dressing changes and at removal to Biobrane.

**Figure 3:**
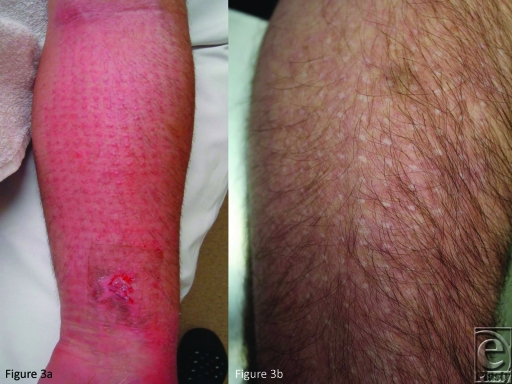
(*a*) By day 21, patient number 3 developed prominences at the site of the AWBAT-S pores. (*b*) When the redness settled from the healed burn, the pore-marks persisted as raised, pale scars.

**Figure 4:**
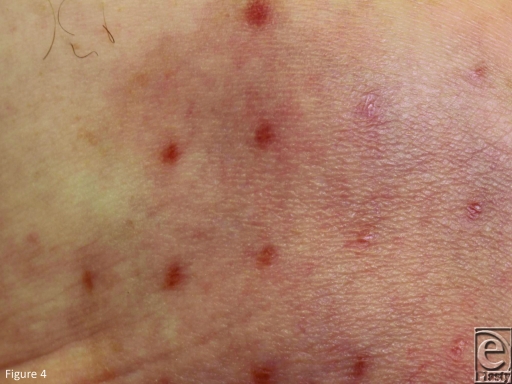
Patient number 10 developed pigmented scars at the pore sites of Biobrane which persisted at 12 months.

**Figure 5:**
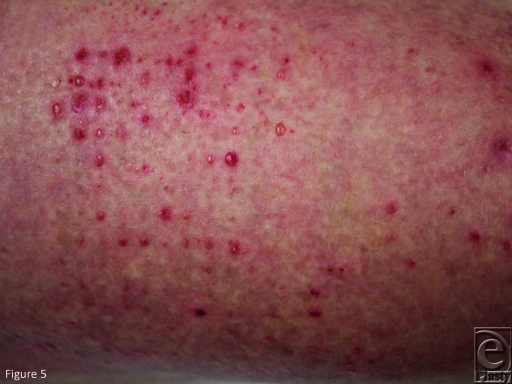
Blistering at AWBAT-S pore sites in patient number 12 followed wearing of nylon track pants within a week of healing.

A final issue relates to pore marks. This is a subject of collaborative investigation close to publication and I do not want to reveal too much about mechanism here; however, pore marks were seen in 5 of the 12 patients at the AWBAT-S treatment area compared with 4 of the 12 Biobrane site pore marks. Patient number 2 developed AWBAT-S pore marks that became prominent between 6 and 12 months and left raised pore scars at 12 months. Patient number 3, had a particularly unusual AWBAT-S pore-mark reaction, with raised prominences that persisted, leaving regular and frequent raised white scars (Figs 3*a* and 3*b*). In all but one patient (number 10), the Biobrane pore marks faded completely (2 by 3 months, 1 by 6 months); patient number 10 had pore scars from Biobrane at 12 months (Fig 4). Patient number 12 completely healed a thigh burn in AWBAT-S without any visible marks, and then developed blisters at the site of the pores after irritation while wearing nylon track pants (Fig 5). These subsequently healed without scarring. It is obvious that the pore-mark phenomenon in Biobrane is not a feature of the discontinuity of the nylon matrix at the pore site, but the discontinuity of the silicone layer. Since AWBAT-S has larger and more frequent pores, the phenomenon appears to persist for longer, and more frequently result in pore scars, than when using Biobrane. Finally, the pink colouration of the healed wound faded more quickly in AWBAT-S–treated areas than Biobrane-treated areas.

## CONCLUSION

AWBAT-S is better than Biobrane in terms of ease of removal and discomfort experienced by patients at this time. For these reasons, the nursing staff preferred it.

AWBAT-S is at least as good as Biobrane in terms of length of hospital stay, time to full healing, and pain/discomfort experienced by patients during healing at rest and therapy. Also, in the general cosmetic appearance of the healed wound under the material proper (not the pores).

AWBAT-S is not as good as Biobrane at the pore sites where a much greater number of AWBAT-S sites displayed pore marks compared with Biobrane. It is not as good as Biobrane in terms of its elasticity or its “‘fix-ability” with adhesive tapes.

Since my practice is about patients, despite my personal happiness with Biobrane, I would consider a change to AWBAT-S if the pores were made comparable in size and frequency to Biobrane (ameliorating the pore-mark issue) and if the issues of reliable availability, regulatory clearance and cost allowed. The material is not so different from Biobrane that I would accept a higher market cost.

## Figures and Tables

**Table 1:** Length of stay, pain scores, time to healing and total body surface area for all patients

		Pain Score Biobrane			Pain Score AWBAT			Time to Healing		
Patient Number	Length of Stay	Day 1	Day 2	Day 3	Day 1	Day 2	Day 3	Biobrane	AWBAT	Total Body Surface Area
1	5	6	4	5	4.5	6	3	12	12	2
2	7	7	7	4.5	8	7	4.5	12	12	5
3	13	0	0	1	0	0	1	10	7	30
4	4	2	1.5	2	2	1.5	3	10	8	10
5	5	3	1	3	2	1	2	7	7	3
6	5	1	1	1	1	1	1	7	7	7
7	10	1	0	3	1	0	3	8	8	11
8	5	0	0	2	0	0	2	13	11	8
9	3	3	2	0	3	2	0	8	8	4
10	7	0	1	3	0	1	2	14	14	5
11	7	0	0.5	1	0	0.5	1.5	10	10	2.5
12	11	8	8	9	8	8	9	11	11	6

**Table 2:** Median and interquartile range for length of stay, pain scores, and total body surface area with mean and standard deviation for time to healing

		Pain Score Biobrane			Pain Score AWBAT			Time to Healing		
	Length of stay, Days	Day 1	Day 2	Day 3	Day 1	Day 2	Day 3	Biobrane	AWBAT	Total Body Surface Area
Mean								10.17	9.58	7.8
SD								2.33	2.4	7.55
Median	6	1.5	1	2.5	1.5	1	2			5.5
Interquartile range	2.75	3.75	2.12	2.38	3.38	2.62	1.62			4.75
